# Vitamin E Levels in Preterm and Full-Term Infants: A Systematic Review

**DOI:** 10.3390/nu14112257

**Published:** 2022-05-28

**Authors:** Débora Gabriela Fernandes Assunção, Lorena Thalia Pereira da Silva, Juliana Dantas de Araújo Santos Camargo, Ricardo Ney Cobucci, Karla Danielly da Silva Ribeiro

**Affiliations:** 1 Post Graduate Program in Nutrition, Health Science Center, Federal University of Rio Grande do Norte, Natal 59078-900, Brazil; deboraassuncao.nutricionista@gmail.com; 2 Graduate in Nutrition, Health Science Center, Federal University of Rio Grande do Norte, Natal 59078-900, Brazil; lorenathaliaps@gmail.com; 3 Maternity School Januário Cicco (MEJC/EBSERH), Federal University of Rio Grande do Norte (UFRN), Natal 59012-310, Brazil; juliana.camargo@ebserh.gov.br (J.D.d.A.S.C.); ricardo.cobucci.737@ufrn.edu.br (R.N.C.); 4 Post Graduate Program of Biotechnology, Potiguar University (UnP), Natal 59056-000, Brazil; 5 Department of Nutrition, Federal University of Rio Grande do Norte, Natal 59078-900, Brazil

**Keywords:** alpha-tocopherol, vitamin E deficiency, neonate, labor

## Abstract

Vitamin E deficiency (VED) is associated with clinical repercussions in preterm newborns (PTN), but low levels are also found in full-term newborns (TN). As this inadequacy can compromise neurogenesis in childhood, studies are needed to assess whether there is a difference in vitamin E status among newborns according to gestational age to provide support for neonatal monitoring protocols. This systematic review presents a synthesis of the available information on the vitamin E status among PTN and TN. The review was performed according to Preferred Reporting Items for Systematic Reviews and Meta-Analyses (PRISMA). Observational studies that evaluated alpha-tocopherol levels were searched in the databases reported in the protocol registered in PROSPERO (CRD42021165152). The Newcastle–Ottawa Scale was used to assess the methodological quality. Overall, 1809 articles were retrieved; 10 were included in the systematic review. In the PTN, the alpha-tocopherol levels ranged from 3.9 to 8.5 mmol/L, while in TN, they were 4.9 to 14.9 mmol/L, and VED ranged from 19% to 100% in newborns. Despite substantial heterogeneity in research methodology and VED classification, the results suggest that the alpha-tocopherol levels among preterm and full-term newborns is below the recommended levels. Our findings demonstrate that further investigations are needed to standardize this classification and to monitor vitamin E status in birth and postnatal with adequate bias control.

## 1. Introduction

Vitamin E is an important antioxidant that occurs in nature in eight different chemical forms, including four forms of tocopherol (α, β, γ, and δ) and four tocotrienols (α, β, γ, and δ), with alpha-tocopherol, a bioactive form, corresponding to approximately 90% of the vitamin found in the body [1,2,3]. Alpha-tocopherol is not produced by our bodies and is predominantly found in peanuts, almonds, and sunflower seeds and oil, while γ-tocopherol is found in walnuts, pecans, pistachios, and sesame seeds. [1,3]. During pregnancy, vitamin E is involved in the development of the embryo, implantation, placental maturation, and protection of the fetus against oxidative stress. During parturition, vitamin E reacts with reactive oxygen species (ROS), thus preventing lipid peroxidation reactions that can occur when the neonate passes to a more hyperoxic environment compared to the uterus [3,4].

Vitamin E deficiency (VED) in newborns has been recorded over time [5]. However, there is a claim that low circulating levels occur only in preterm newborns (PTN) (<37 weeks at birth) [5,6] because of the limited placental transfer of the vitamin due to the high oxidative stress during delivery and the immaturity of neonatal lipid metabolism and the antioxidant defense system [7,8,9]. Low alpha-tocopherol concentrations in newborns are associated with the development of intraventricular hemorrhage, bronchopulmonary dysplasia, central nervous system developmental delay, ataxia, myopathy, edema, thrombocytosis, and hemolytic anemia, which can result in spinocerebellar degeneration [2,10] and cardiomyopathy as a consequence of probable muscle degeneration [6,7,11].

From this perspective, some studies have shown that low levels of vitamin E can also be found in full-term newborns (TN), but this information has not been explored in the literature [12,13]. Recently, vitamin E status has been associated with neurogenesis and cognition, possibly due to the protective effects of vitamin E against oxidative stress damage and its suppressive role in the expression of many genes involved in neurodegeneration [11]. Traber et al. [14] verified that vitamin E plays an important role during neuroembryogenesis and demonstrated that VED causes metabolic dysregulation and impacts morphological changes in the early stages of development. In fish embryos, VED causes metabolic disturbances and impacts the early developmental stages [9], highlighting the importance of adequate vitamin E levels during pregnancy and birth.

Therefore, as vitamin E is essential in biological processes, and its deficiency can cause long-term damage to infants, resulting from negative outcomes in newborns further affecting childhood cognition [11], it is necessary to assess whether there is a difference in the nutritional status of vitamin E between TN and PTN. The results can provide information that facilitates planning and evidence-based decision-making, with a focus on preventive actions and health promotion to avoid possible VED-induced damage to neonatal health. Thus, the objective of this study was to compare the nutritional status of vitamin E between PTN and TN.

## 2. Materials and Methods

This systematic review was performed according to the Preferred Reporting Items for Systematic Reviews and Meta-analyses (PRISMA) guidelines [15] and registered in the International Prospective Register of Systematic Reviews (PROSPERO) under the number CRD42021165152.

The following research question was addressed: Is there a difference in the nutritional status of vitamin E between term and preterm newborns? It was formulated according to the PICoS structure, which consists of addressing the population, interest, context, and study design. We implemented the PICoS description as follows:

(1) Population (P): newborns; (2) Interest (I): vitamin E nutritional status (serum/plasma alpha-tocopherol); (3) Context (Co): premature newborns and term newborns; and (4) Study design (S): cohort, cross-sectional, and case-control studies.

The search for studies was performed using the PubMed/Medline, EMBASE, Web of Science, and SciELO databases. Additional methods were included, such as a citation search, a search in the references of selected articles and a search in the depository of dissertations and theses, in order to expand the search. Data collection, analysis, and extraction were performed by two independent researchers (D.G.F.A. and L.T.S.), and disagreements were resolved by a third reviewer (K.D.S.R.)

The search strategy contemplated the combination of Medical Subject Headings (MeSH) terms with entry terms: “Infants, Newborn”; “alpha-tocopherol”; “vitamin E deficiency”; “infant, premature”, as shown in Table 1. During the searches, filters were not added to the research bases, which led to it reporting a large number of articles without the eligibility criteria for inclusion in this review.

### 2.1. Inclusion and Exclusion Criteria

The study population consisted of newborns classified according to the World Health Organization (WHO) [16], with preterm newborns being those with a gestational age of <37 weeks at birth, and full-term newborns being those with a gestational age ≥37 weeks. Only observational studies with data on serum/plasma alpha-tocopherol reported umbilical cord levels of preterm and/or full-term newborns were considered. Studies (a) with multiple gestations, (b) in animal models, (c) that did not analyze serum alpha-tocopherol, and/or (d) did not use high-performance liquid chromatography (HPLC) for the determination of alpha-tocopherol were excluded.

### 2.2. Study Quality Analysis

All selected articles were assessed for quality using the Newcastle–Ottawa Scale (NOS) [17,18,19], according to the following domains: patient selection (generalization and applicability), comparability of study groups, methods for evaluating outcomes (cohort studies), proof of exposure (case-control), and adequate follow-up.

### 2.3. Data Extraction

Data extraction was performed by two authors (D.G.F.A. and L.T.S.) in an electronic spreadsheet containing the following fields: authorship, year of publication, country of study, sample number, gestational age, alpha-tocopherol analysis method, alpha-tocopherol levels in the groups, vitamin E deficiency (%), cutoff point used for VED, use of maternal supplementation, factors related to alpha-tocopherol, and assessment of study quality. The umbilical cord levels of alpha-tocopherol were converted into µg/dL as a form of standardization. The cutoff point for VED was used according to the reference described in the article, and the associated factors according to the results of the regression or correlation analyses that correlated with alpha-tocopherol in the studies. When more than one publication of the same study was found, data from the most complete publication were extracted. For abstracts not found in full, the authors were consulted about the possibility of making the complete material available. When not available, we tried directly contacting authors.

### 2.4. Sensitivity Analysis

We performed a sensitivity analysis separating studies with poor (up to 4 stars), good (between 5 and 6 stars), and excellent (7 stars) methodological quality according to the NOS. For the meta-analysis, the I² test was used, with a result equal to 77%, demonstrating high heterogeneity (>50%) in the studies found. Thus, it was not possible to perform a meta-analysis in this study.

## 3. Results

**Figure 1:**
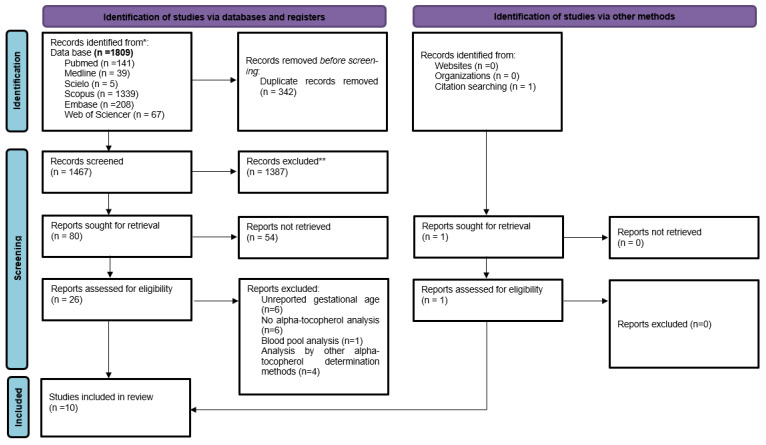
PRISMA flow diagram.

Initially, 1809 articles were obtained using the search strategy, 342 duplicates were excluded, and 1467 studies were selected for the analysis of titles and abstracts. After this step, 80 articles were selected for complete reading of the text, with 10 studies included in the systematic review [20,21,22,23,24,25,26,27,28]. Figure 1 shows the study selection flowchart.

### 3.1. Study Characteristics

Of the ten articles included in the review [8,20,21,22,23,24,25,26,27,28], only one was not found in full for complete reading [20]; however, it was included in this review to present the information in its abstract, including the mean alpha-tocopherol concentration and standard deviation.

Four studies were cohorts [20,25,26,27], and only seven articles specified the study’s country of origin: two were in Africa [22,28], two were in the United States [8,26], one was in Brazil [25], and two were in Europe [22,24]. Table 2 presents the main information of the studies.

### 3.2. Vitamin E Status Analysis and Associated Factors

There was an assessment of alpha-tocopherol levels in TN and PTN in only five studies [20,21,24,25,28]. In the PTN group, the umbilical cord blood alpha-tocopherol levels ranged from 3.9 mmol/L [20] to 8.5 mmol/L [27], while in TN, the values ranged from 4.9 mmol/L [20] to 14.9 mmol/L [28]. The greatest variation found between the TN and PTN groups was in a study by Ghany et al. [28], where the mean concentrations in the PTN and TN groups were 5.2 mmol/L and 14.9 mmol/L, respectively. Only two articles clarified that women did not use vitamin E supplements during pregnancy [22,25], and one cited this supplementation.

Of the five studies that analyzed vitamin E levels in both TN and PTN [20,21,24,25,28], only one found a significant difference between the groups, with vitamin E concentrations being lower in the PTN group [28].

Regarding the variables associated with alpha-tocopherol in the umbilical cord, one study associated it with circulating lipids [20], two with gestational age [21,27], one with hemolysis [27], one with circulating triglycerides [22], one with income [24], two with maternal alpha-tocopherol [8,25], and one with postpartum time [28].

### 3.3. Vitamin E Deficiency (VED)

Only three articles assessed VED in the studied population [20,21,25], but different cutoff points for VED classification (<3.5 mmol/L to <11.6 mmol/L of alpha-tocopherol) were used.

In two studies [20,25], the cutoff point was 11.6 mmol/L to assess VED, resulting in a prevalence between 92% and 100% regardless of the gestational age at delivery. Chan et al. [21], using a lower cut-off point of <3.5 mmol/L for alpha-tocopherol, found VED in 38% of their PTN population and 19% of the TN population.

### 3.4. Methodological Quality

The overall average of the studies on the NOS was 5.5 stars, with a grade of 7 being the highest score achieved (*n* = 2), and 2 the lowest (*n* = 1). In the selection domain, there was a low score in relation to the representativeness of the sample. The case-control study had lower scores, especially in the selection domain. The methodological quality of the studies is presented in Table 2.

## 4. Discussion

Vitamin E deficiency or inadequacy in newborns requires further study. It is rare in adults, but it has been registered over time in newborns [6,7]. However, there is a debate on whether low circulating levels of vitamin E in newborns can be a transitory and physiological situation only at birth, and whether it affects only PTN [8,29]. In this systematic review, there was no significant difference in vitamin E levels between TN and PTN in most studies.

Among the five studies that compared vitamin E levels in TN and PTN [20,21,24,25,28], only one found a significant difference between the groups, with vitamin E concentrations being lower in preterm infants and the highest values in the full-term compared to the other studies [28]. This case-control study was conducted with 200 newborns, 100 of whom were TN and 100 who were PTN, with the gestational age of the latter ranging from 27 to 34 weeks of gestation, not including newborns classified as late preterm (>34 weeks). The elevated variability of alpha-tocopherol levels in PTN (3.9–8.5 mmol/L) and TN (4.9–14.9 mmol/L), the inclusion of extremely preterm infants (<28 weeks) and the exclusion of late preterm infants may be some of the possible causes for this difference, since these newborns may be more susceptible to factors, such as the limited placental transfer of vitamin E to the fetus [9].

Vitamin E deficiency was analyzed only in three studies included in this review, with each using different cutoff points to define VED or inadequacy (<3.5 mmol/L to <11.6 mmol/L) [20,21,25], and a high prevalence of VED was found. In this review, the lowest prevalence of VED was found in a study that used the lowest cutoff point (<3.5 mmol/L) [21], a fact that may justify the values found.

These lower alpha-tocopherol values at birth in both term and preterm infants can be hypothesized to be influenced by some factors: (1) It is known that newborns have one-third of the maternal serum alpha-tocopherol [25], suggesting that, naturally, there will be lower alpha-tocopherol concentrations in the umbilical cord, probably due to the lower placental activity of the alpha-tocopherol transfer protein (α-TTP) in babies [30]; (2) There is high oxidative stress at the time of delivery due to the newborn’s exposure to a hyperoxic environment, which can reduce vitamin E levels in the newborn [9]; (3) The consumption of vitamin E during pregnancy is low [31]; (4) An adequate intake of vitamin E during pregnancy can favor the maintenance of appropriate circulating vitamin E levels and minimize the toxic effects of oxidation products in the newborn [32].

Although VED in newborns has been described for some time, it is clear that there is no consensus on how to make this diagnosis, how to monitor it, or the magnitude of the problem. The concentration of alpha-tocopherol in umbilical cord serum or the general circulation has been studied as the main biomarker of vitamin E nutritional status in PTN [33], but there is still a debate on whether low alpha-tocopherol values at birth are indicative of a deficiency or an inadequacy [5,25,26]. A cutoff point of <11.6 mmol/L of alpha-tocopherol for the classification of VED was proposed by Gutcher [26], who verified that lower vitamin E concentrations promoted peroxide-induced hemolysis in an in vitro study. The cutoff point of <3.5 mmol/L of alpha-tocopherol used by Chan was taken from a book published in 1992, access to which is not available, making it impossible to understand the mechanism by which this value was proposed [21]. The ESPGHAN/ESPEN/ESPR/CSPEN guidelines recognize normal values for serum tocopherol between 100 and 200 µg/dL to treat the clinical repercussions of VED [34].

Given the different cutoff values used for vitamin E deficiency, further studies are needed to standardize this classification and to better understand the status of this vitamin at birth, as both PTN and TN groups seem to have low levels when analyzed using the existing cutoff points. However, studies point to an increase in alpha-tocopherol concentrations after birth, which suggests a transient deficiency/inadequacy of vitamin E in newborns. Furthermore, it is important to emphasize that this increase does not seem to occur at satisfactory levels in all newborns [6,25,27]. In addition, this increase in serum alpha-tocopherol appears to occur progressively in infants who are exclusively breastfed during the first, second, and third months of life, while these values progressively decrease in formula-fed infants, thus demonstrating the importance of breastfeeding in increasing levels of alpha-tocopherol over the first few months [27]. Rodrigues [25] also found similar results, verifying that at 90 days after birth, regardless of the gestational age at birth, alpha-tocopherol concentrations were higher in those who were exclusively breastfed (14.0 ± 5.3 mmol/L) than in those who were not (517.5 ± 100.4 µg/dL).

Thus, this evidence emphasizes the importance of breastfeeding to increase infant serum alpha-tocopherol concentrations, reinforcing the need to encourage and support breastfeeding for the proper development of the newborn by aiding neurological development and cognition [25]. Therefore, breastfeeding offered to newborns whose vitamin E levels were measured in the studies included in this review may have influenced the lack of difference in nutritional status found (in the systematic review).

It is important to emphasize that there are limitations in this review, such as the absence of sample calculation in several studies; a lack of statistical information, such as mean dispersion measures; the use of different sample sizes when evaluating PTN and TN; serum vitamin E assessment conducted only in one group; and the absence of confounding factor evaluation that may be related to VED, such as level of prematurity, social characteristics, ethnicity, breastfeeding time, maternal nutritional status/diet, infant nutritional status at birth [BW/IUGR/SGA], type of delivery, and use of vitamin E-containing supplements during pregnancy. From this perspective, although vitamin E supplementation in neonates increases its concentrations and oral vitamin E supplementation effectively prevents the development of retinopathy in very low birth weight infants with respiratory distress syndrome, this strategy is not recommended because of the risk of sepsis that supplementation with high doses of vitamin E can cause in newborns [33]. Thus, exclusive breastfeeding is an efficient strategy to increase vitamin E reserves in newborns [9], because when lactating women are supplemented with vitamin E in its natural form (RRR-alpha-tocopherol), an increase in vitamin E concentrations in colostrum and mature milk can be seen [35,36].

This systematic review brings important contributions to neonatal healthcare because the results suggest that it is possible to find low levels of alpha-tocopherol in both PTN and TN in birth. Untreated VED is related to neurological and cognitive development disorders throughout childhood; therefore, it is important to monitor vitamin E concentrations, especially in low-birth-weight infants and infants not exclusively breastfed, as breastfeeding seems to guarantee an increase in vitamin E levels in the postnatal phase [25]. However, the absence of quality studies that allow us to say that there is a difference in vitamin E levels between PTN and TN means that more studies should be conducted to verify the vitamin E status in the postnatal phase according to breastfeeding type and with control for confounding variables, such as clinical complications, socioeconomic conditions, and the mother’s diet.

## Figures and Tables

**Table 1:** Search equation to perform the review. July/2021.

Search Equation
Population: “Infants, Newborn” [MESH terms] Neonate OR Neonates OR Newborn OR Newborn Infant OR Newborn Infants OR Newborns
AND
Interest: “Alpha-tocopherol” [MESH terms] (Alpha-tocopherol OR R,R,R-alpha-Tocopherol OR Tocopherol Acetate OR Tocopherol Succinate OR Tocopheryl Acetate OR Vitamin E Succinate OR alpha-Tocopherol Acetate OR alpha-Tocopherol Hemisuccinate OR alpha-Tocopherol Succinate OR alpha-Tocopheryl Calcium Succinate OR d-alpha Tocopherol OR d-alpha-Tocopheryl Acetate)
AND
Context: “Infant, Premature” [MESH terms] (Infant, Preterm OR Infants, Premature OR Infants, Preterm OR Neonatal Prematurity OR Premature Infant OR Premature Infants OR Prematurity, Neonatal OR Preterm Infant OR Preterm Infants) “Infants, Newborn” [MESH terms] Neonate OR Neonates OR Newborn OR Newborn Infant OR Newborn Infants OR Newborns “Deficiency vitamin E” [MESH terms] “Deficiencies, Vitamin E or Deficiency, Vitamin E or Vitamin E Deficiencies”

MeSH, Medical Subject Headings.

**Table 2:** Characteristics of the studies included in the systematic review.

Author	Study Design	Country	*n*	Gestational Age (Weeks)	Analysis Method	Alpha-Tocopherol (mmol/L) in the Umbilical Cord Blood *** Mean ± Standard Deviation		% VED	VED Cutoff (mmol/Ll)	Use of Maternal Supplement	Factors Related to Alpha-TOH	QualityAssessment **
						Preterm	Full-Term					
Wu; Chou [20] (2001)	Cross-Sectional	NA	35 PTN 34 TN	PTN: 28 to 34 TN: 38 to 42	HPLC	3.9 ± 2.1	4.9 ± 2.9	PTN: 100% TN: 94%	<11.6	NA	Circulating lipids	5
Chan et al. [21] (1999)	Cross-Sectional	NA	40 PTN 180 TN	NA	HPLC	6.1 *	6.4 *	PTN: 38% TN: 19%	<3.5	NA	Gestational Age and tocopherol in the umbilical cord	4
Gonzalez-Corbella et al. [24] (1998)	Cohort	Spain	8 PTN 48 TN	PTN: 34 to 36 TN: >37	HPLC	8.2 ± 2.2	12.1 ± 7.3	NA	NA	NA	Income	6
Rodrigues [25] (2016)	Cohort	Brazil	124 PTN 111 TN	PTN: <37 TN: >37	HPLC	6.4 ± 3.1	5.5 ± 3.0	PTN: 94% TN: 92%	<11.6	None	Maternal alpha-tocopherol	7
Ghany et al. [28] (2016)	Case-Control	Egypt	100 PTN 100 TN	PTN: 27 to 34 TN: 37 to 40	HPLC	5.2 *	14.9 *	NA	NA	NA	Gestational age	7
Kiely et al. [22] (1999)	Cross-Sectional	Ireland	40 PTN	NA	HPLC	NA	7.2 *	NA	NA	None	Circulating Triglycerides	5
Gutcher [26] (1984)	Cohort	USA	62 PTN	31	HPLC	5.8 ± 2.5	NA	NA	NA	NA	Hemolysis	6
Kaempf et al. [27] (1998)	Cohort	NA	14 PTN	30	HPLC	8.5 ± 3.0	NA	NA	NA	NA	Gestational age and alpha-tocopherol in the umbilical cord	5
Okolo et al. [23] (1989)	Cross-Sectional	Nigeria	25 TN	37 to 42	HPLC	NA	11.1 ± 4.7	NA	NA	NA	NA	5
Didenco et al. [8]	Cross-Sectional	USA	19 TN	≥38	HPLC	NA	6.7 ± 2.5	NA	NA	Yes	Maternal alpha-tocopherol (after adjustment for total lipids)	2

NA—Not evaluated/presented. * No standard deviation. ** Newcastle–Ottawa Scale. *n*—sample number. *** All studies used umbilical cord serum for alpha-tocopherol analysis. VED—Vitamin E deficiency. PTN—Preterm newborn. TN—Term newborn. Alpha-TOH—Alpha-tocopherol.

## Data Availability

Not applicable.
